# EMG amplitude of the biceps femoris during jumping compared to landing movements

**DOI:** 10.1186/2193-1801-2-520

**Published:** 2013-10-09

**Authors:** Johnny Padulo, Alessandra Tiloca, Douglas Powell, Giampietro Granatelli, Antonino Bianco, Antonio Paoli

**Affiliations:** Faculty of Medicine & Surgery, University of “Tor Vergata”, Rome, Italy; Tunisian Research Laboratory “Sports Performance Optimization”, National Center of Medicine & Science in Sport, Tunis, Tunisia; CONI – Italian Regional Olympic Committee, Sardinia, Italy; Department of Physical Therapy, College of Pharmacy & Health Sciences, Campbell University, Buies Creek, NC USA; Sport & Exercise Sciences Research Unit, University of Palermo, Palermo, Italy; Department of Biomedical Sciences, University of Padova, Padova, Italy

**Keywords:** Braking phase, Counter movement jump, Squat jump, Electromyography, Stretch-shortening cycle, Hamstring injuries

## Abstract

Hamstrings injury is a common occurrence in athletic performance. These injuries tend to occur during a deceleration or landing task suggesting the negative work may be a key component in hamstrings injury. The purpose of this study was to investigate the muscular activity (EMG) of the biceps femoris (BF) in different phases (concentric vs. eccentric) of a Counter Movement Jump (CMJ), Squat Jump (SJ) and the Braking Phase (BP) of a landing task. Twelve female volleyball players performed 5 CMJs, SJs and BPs while surface EMG was recorded using a MuscleLab (BoscoSystem^TM^, Norway). EMG values were normalized to an maximal voluntary contraction. A repeated measures analysis of variance (ANOVA) was used to compare mean normalized EMG values of the concentric and eccentric portions of the CMJ with the BP and SJ. The ANOVA revealed significantly lower BF activation in the concentric and eccentric portions of the CMJ compared to the BP (64%, *p* < 0.001) and SJ (7%, *p* = 0.02), respectively. These findings suggest that the CMJ relies on a greater contribution of elastic tissues during the concentric and eccentric portions of the movement and thus requires less muscle activation of the BF.

## Introduction

Most athletic movements are multi-joint in nature and require precise and powerful movement of a number of segments through a range of motion. Muscular contraction is responsible for the initiation and control of segmental motions. These muscular contractions can be classified by changes in overall muscle length during force production (Bosco et al. 1982a). Concentric muscle contraction is characterized by an overall shortening of the muscle length during force production (Padulo et al. 2013) while eccentric muscle contractions can be described as lengthening contractions during which the overall length of the muscle becomes longer during force production (Padulo et al. 2013). In athletic movements it is common that a shortening contraction is performed immediately following muscle stretch, termed stretch-shortening cycle (Bosco et al. 1981; Padulo et al. 2013). Previous research has demonstrated that more work is performed in a stretch-shortening cycle compared to a purely shortening contraction (Ciullo and Zarins 1983). Cavagna et al. (Cavagna et al. 1965; Cavagna et al. 1968) stated that pre-stretch of an active muscle is associated with energy stored within the elastic components of the muscle-tendon unit and is released during the concentric (shortening) phase of the stretch-shortening cycle. However, more recent research has demonstrated that the quantity of energy released during the shortening phase is greater in magnitude than the energy stored in the elastic components (Ciullo and Zarins 1983). Changes in the intrinsic mechanical properties of myofibrils have been suggested as a potential source of enhanced performance associated with the stretch-shortening cycle (Takarada et al. 1997).

The stretch-shortening cycle is often used in athletic movements and training to enhance performance (Bosco et al. 1982a). Common clinical measures of lower extremity power and performance include the squat jump (SJ), counter movement jump (CMJ) and drop jump (DJ). There are distinct differences between these three movements with regard to the types of muscle contractions required for successful completion. Specifically, the squat jump is initiated from a position of knee and hip flexion (approximately 90°) and completed by concentric contraction of the hip and knee extensors to propel the individual upward (Padulo et al. 2013). Conversely, the CMJ and DJ movements begin with the participant in an extended position as the individual is standing or landing from a height (DJ). In these movements the individual performs rapid flexion movements via eccentric (lengthening) contractions of the hip and knee extensors, increasing the length of these muscles, followed by rapid concentric (shortening) muscle contraction of the hip and knee extensors (Padulo et al. 2013). Conversely, in a landing the lower extremity musculature contracts eccentrically to attenuate kinetic energy associated with the momentum of the body moving downward due to gravity.

In each of these movements (SJ, CMJ, DJ and landing), the primary hip extensor should be the gluteus maximus, which is a powerful, uni-articular muscle. However, as the population has progressed from a labor-intensive to corporate lifestyle over time, many individuals have developed “gluteal amnesia” (McGill 2007). In individuals with gluteal amnesia, movements that are typically accomplished with the gluteal complex are conducted by shifting the impetus of the movement to the hamstrings and muscles of the low back. Recent research has suggested that the hamstrings musculature has the highest rate of injury of lower extremity musculature (Petersen et al. 2011; Thorborg et al. 2011), and that these injuries occur most frequently during negative work associated with deceleration (Petersen et al. 2011; Woods et al. 2004). The high rate of hamstring injuries may be associated with the exaggerated load placed on these muscles in response to diminished gluteal function or gluteal amnesia. Given the high rate of hamstring injuries in sport, a need emerges to determine the role of the hamstrings in athletic movements involving shortening and lengthening contractions as well as the stretch-shortening cycle.

Therefore, the purpose of this study was to investigate the role of the hamstrings musculature during dynamic loading tasks with different contraction types (concentric vs. eccentric vs. stretch-shortening cycle). It was hypothesized that: (1) the hamstrings musculature would exhibit greater muscle activation in concentric compared to eccentric contractions, and (2) that the hamstrings would exhibit greater muscle activation in the stretch-shortening cycle compared to concentric contractions.

## Methods

### Subjects

Twelve female volleyball players (age 24.7 ± 3.9 years; body mass 60.1 ± 7.6 kg, height 1.68 ± 0.09 m; BMI 21.1 ± 2.0 kg/m^2^) participated in this study. Each participant was an elite level volleyball player, with 9 or more years of experience, and was competing for a national championship at the time of this investigation. All participants were healthy with no musculoskeletal or neurological conditions at the time of participation. The experimental protocol was approved by the Institutional Review Board of the University. Informed consent was obtained from all participants.

### Protocol

**Figure 1:**
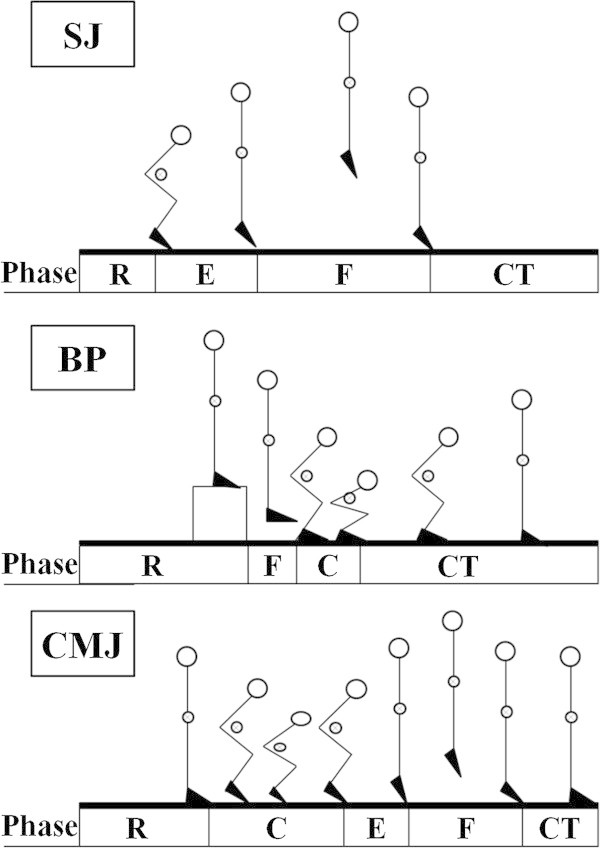
**Representative Squat Jump (SJ), Braking Phase (BP) and Counter Movement Jump (CMJ).** For each jump the description in different phase: ready (R), eccentric phase (E), concentric phase (C), flight phase (F), contact phase (CT).

All participants were in good health at the time of the study and completed testing during the sport season. All data were acquired on a single day. The experimental protocol consisted of completing three successful trials in each of three conditions: Braking Phase of Landing (BP), Counter Movement Jump (CMJ) and Squat Jump (SJ). Subjects were given two minutes of rest between trials within each condition while experimental conditions were randomized using a Latin Square design. The BP trials were characterized by the subject performing a step off landing from a height of 45 cm and adopting a stable, bilateral stance with knee flexion equal to 90°. CMJ trials began with the subject in an upright standing posture, making a preliminary downward movement through knee and hip flexion followed by an immediate, forceful extension of the knee and hip joints propelling the subject vertically off of the ground (Bosco et al. 1981). The SJ started from a stationary semi-squatted position with the knee joint flexed to 90° and involved only the knee extension (Earp et al. 2010). The position of the upper body was also standardized to minimize body flexion and extension of the trunk (Figure 1). This Investigation was approved from the board of CONI “Italian Regional Olympic Committee, Sardinia, Italy” project nr.6; A2 – 2011.

EMG activity of biceps femoris (BF) was used to represent hamstrings muscle function. EMG was recorded during each phase with a sampling frequency of 1500 Hz (MuscleLab™ BoscoSystem, Norway). An amplifier with gain × 600, input impedance 2 GΩ, common-mode rejection ratio 100 dB; (Biochip Grenoble, France) was used. Subjects were asked to contract their muscle against manual resistance and the belly of the muscle was palpated. The skin over the belly of the muscle was shaved and cleansed with alcohol to reduce the skin impedance to 5 kΩ. Bipolar surface electrodes (inter-electrode distance 1.2 cm) were aligned with the longitudinal axis of the muscle according to the recommendations of SENIAM (Hermens et al. 2000). Electrode placement was confirmed using manual muscle testing. To prevent movement artifact, EMG cables were secured to the subject using elastic bands (Vetrap™) (Bosco et al. 1995). Raw EMG signals were rectified and smoothed using the root mean squared (RMS). A Linear Encoder (MuscleLab^TM^ BoscoSystem, Norway with 0.001-m accuracy) was used to monitor subject movements. The Linear Encoder was connected to each subject and interfaced with the computer. All equipment (EMG signals and the biomechanical parameters) were sampled simultaneously using a trigger. Prior to testing, maximal voluntary isometric contractions (MVC) were performed to determine maximal EMG activation for EMG normalization and used for statistical analysis (Bosco and Viitasalo 1982). MVC for biceps femoris was determined by averaging the integrated EMG signal during a 1-s period of sustained maximal force output after the initial peak in the force curve during the isometric squat (with knee angle 90°) (Cormie et al. 2010). Normalized EMG values were calculated as the quotquotient of the recorded EMG from dynamic trials divided by the EMG values recorded from the MVC trials and are represented as a percentage of MVC.

All testing sessions were performed in an area of 1.0 m × 1.0 m, and kinematics were recorded using a high-speed camera (210 Hz, Casio Exilim FH20, Japan) with a resolution of 0.17 MPx. The high-speed camera was placed 2 m from subjects, perpendicular to the plane of motion to record sagittal plane motion, while the camera was accurate to 3 mm (Belli et al. 1992). The flight/contact phases (milliseconds) were calculated the number of frames on the *2D* data, divided by the sampling rate, 210 (1 frame = 210 Hz ≈ 0.0048 s). The film sequences were analyzed off-line using Dartfish 5.5 Pro motion analyzing software (Dartfish, Fribourg, CH).

### Statistical Analyses

Four Univariate analyses of variance (ANOVAs) were used to determine significant differences in EMG amplitude and movement duration between movements, for each phase (concentric vs. eccentric). Specifically, the ANOVAs were used to compare variables of interest during the concentric phases of Landing and CMJ trials as well as the eccentric phases of the CMJ and SJ. A Student’s *t*-test was used to determine significant differences in flight time between the CMJ and SJ movements as a measure of overall performance. All statistical analyses were conducted using SPSS 15.0 (IBM Corp., Somers, NY, USA). Alpha level was set at *p* < 0.05.

## Results

**Figure 2:**
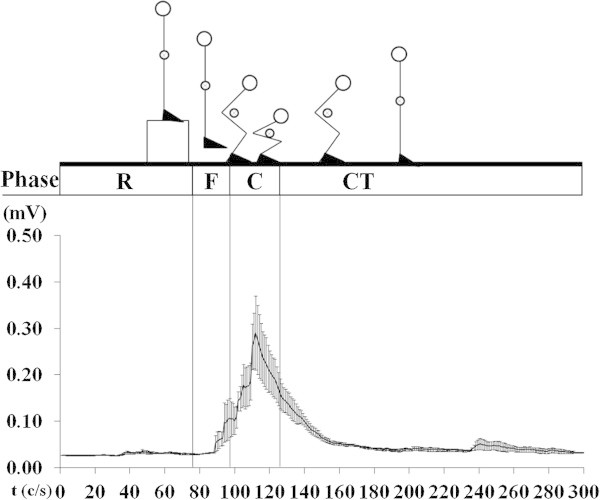
**Rectified and smoothed EMG curves indicate electrical activity of the biceps femoris during Braking Phase, all synchronized with different phase: ready (R), flight (F), concentric (C), and contact time (CT).**

**Figure 3:**
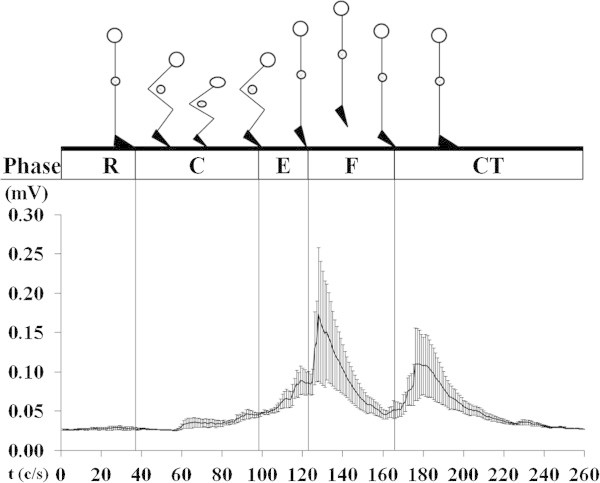
**Rectified and smoothed EMG curves indicate electrical activity of the biceps femoris during Counter Movement Jump, all synchronized with different phase: ready (R), concentric (C), eccentric (E), flight (F) and contact time (CT).**

**Figure 4:**
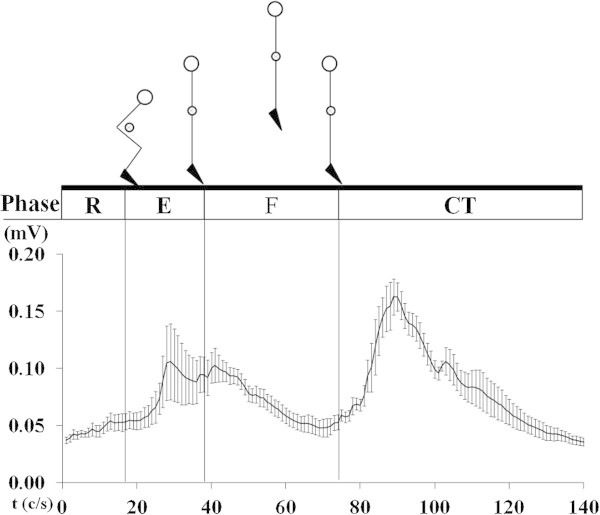
**Rectified and smoothed EMG curves indicate electrical activity of the biceps femoris during Squat Jump, all synchronized with different phase: ready (R), eccentric (E), flight (F) and contact time (CT).**

**Figure 5:**
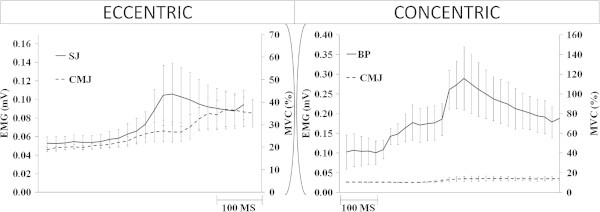
**Rectified and smoothed EMG curves indicate electrical activity of the biceps femoris during: eccentric phase (Squat Jump (SJ) vs. Counter Movement Jump (CMJ)) and concentric phase (Braking Phase (BP) vs. CMJ).** The EMG was divided in relation to eccentric vs. concentric phase. The dashed horizontal lines (right) indicate the maximum (100%) EMG measured during maximal voluntary contractions (MVC) while the horizontal lines (left) indicate EMG activity in mV.

The braking time of the concentric phase was shorter (*p* = 0.004) in BP (306 ± 82.80 ms Figure 2) compared to CMJ (495 ± 149 ms Figure 3). EMG amplitude of the BF was greater (*p* < 0.0001) in the BP (76% MVC) compared to CMJ (Figure 4) 0.03 ± 0.01 mV (12% MVC). During the eccentric phase, the SJ (219 ± 30.29 ms Figure 4) had less propulsive time (*p* < 0.05) than the CMJ (262 ± 64.80 ms). EMG activity (Figure 5) was greater (*p* = 0.02) in the SJ (0.08 ± 0.02 mV; 32% MVC), compared to the CMJ (0.07 ± 0.01 mV; 25% MVC) No differences (*p* = 0.673) were observed in flight time between the SJ (386 ± 27.03 ms) and CMJ (392 ± 28.38 ms).

## Discussion

The purpose of this study was to quantify BF activation during jumping and landing activities with specific focus on the mode of contraction. The current findings demonstrate that the biphasic coupling of concentric and eccentric contractions during the stretch-shortening cycle results in significant reductions in BF muscle activation. The muscle-tendon unit is comprised of the contractile component of muscle and the surrounding connective tissues that make up the parallel and series elastic components (Bosco et al. 1981; Bosco et al. 1982c; Bosco et al. 1982b). The muscular component is associated with active force production in response to electrical stimulus from the nervous system (Bosco et al. 1982a). Conversely, the elastic components of the muscle-tendon unit are passive in nature and have been modeled mechanically as springs. Previous research has demonstrated that muscles can produce more positive work (Cavagna and Citterio 1974; Fenn 1924) and power (Padulo et al. 2012) when the muscle is actively stretched prior to shortening (stretch-shortening cycle). Several mechanisms have been proposed to underlie the observed improvements in mechanical performance associated with the stretch-shortening cycle including increased time to develop force, storage and release of elastic energy, force potentiating and reflexes (Enoka 2008).

The focus of this study pertains to the storage and release of elastic energy in contrast to the facilitation of muscle activation by the stretch reflex. The elastic nature of the connective tissues suggests that as the tendon is stretched, the mechanical energy acting to stretch the tendon is stored as potential energy which is then released as the tendon is shortened (Enoka 2008). It has been suggested that the release of passive, elastic energy during the concentric phase of movements that contain an eccentric-concentric stretch-shortening cycle results in improved mechanical performance (Henry et al. 2005; Ishikawa et al. 2006; Kawakami et al. 2002; Kubo et al. 2007; Kurokawa et al. 2003).

In addition to the kinetic advantages of the stretch-shortening cycle, it has been suggested that stretch of the muscle-tendon unit enhances the efficacy of neuromuscular activation (Bosco et al. 1981). Specifically, the muscle spindle is activated in response to muscle stretch and increases alpha motor neuron activation resulting in activation of the muscle being stretched (Enoka 2008). The intensity of muscle activation in response to stretch is proportional to the rate and magnitude of muscle stretch (Matthews 1991). Accompanying the agonistic stretch reflex is reciprocal inhibition (Hultborn 2006; Sherrington 1897). Reciprocal inhibition links the inhibition of antagonistic muscle activation to agonist muscle activation, thereby reducing the intensity of the antagonistic muscle activation and improving performance (Hultborn 2006; Sherrington 1897). Previous research has suggested that the stretch reflex may be a source of improved performance in a stretch-shortening cycle compared to a solely shortening contraction (Avela et al. 2006; Nicol and Komi 1998). More specifically, Kawakami et al. (2002) investigated the differences in muscle activation and force production during a counter movement jump and squat jump (Kawakami et al. 2002). The counter movement jump was associated with smaller EMG amplitudes of the gastrocnemius and greater peak ground reaction forces than the squat jump (Kawakami et al. 2002). The findings of the present study support the findings of Kawakami et al. (2002) and demonstrate greater EMG amplitudes of lower extremity musculature in the squat jump compared to the counter movement jump (Kawakami et al. 2002). Though Kawakami et al.’s study focused on the gastrocnemius and the present study investigated the biceps femoris, both muscles are large anti-gravity muscles responsible for vertical propulsion during the jumping movement (Kawakami et al. 2002). Both the gastrocnemius and biceps femoris are biarticular muscles crossing the knee joint and would experience stretch during the countermovement jump while the stretch reflex would not be elicited during the squat jump. The findings of both the present study and Kawakami et al.’s study reveal reduced neuromuscular activation in the counter movement compared to squat jumps suggesting that the fewer muscle fibers are being activated to perform the jumping task (Kawakami et al. 2002). The current data also demonstrated that muscular performance, measured by vertical jump height, was not significantly different between the counter movement and squat jumps. Therefore, it can be concluded that the counter movement jump is associated with improved mechanical efficiency and that this enhanced efficiency is the result of increased elastic contribution to the jumping movement.

A central component in the improved jumping efficiency of the counter movement jump compared to the squat jump is the eccentric muscle loading and stretch of the elastic components of muscle prior to the concentric jumping movement. To the knowledge of the authors, no previous research study has compared muscle activations of the biceps femoris in a purely concentric, landing movement and a counter movement jump. The findings of this study revealed that muscle activation is significantly greater in a purely eccentric, landing movement compared to the counter movement jump. The improved muscular efficiency of the jumping portion of the counter movement jump has been attributed to the potential energy stored in the elastic tissues during the downward phase prior to the jumping movement. However, the present data also demonstrate that the eccentric component of a landing task is associated with significantly greater muscle activation than the eccentric phase of the counter movement jump. It can be postulated that the reduced muscle activation associated with the counter movement jump is the result of the release of the elastic energy during the counter movement jump while in the landing task the kinetic energy of landing was attenuated via eccentric muscular contraction rather than elastic tissues resulting in greater EMG amplitudes. Interestingly, the duration of the eccentric component of the counter movement jump (495 ms) was significantly greater than during the landing task (306 ms). The greater duration of the eccentric component of the counter movement jump may also underlie the reduced EMG amplitudes as the kinetic energy was attenuated over a longer period of time requiring smaller peak muscular forces.

The high incidence of hamstring injuries in athletics can be attributed to a combination of muscular and mechanical factors including muscle activation and external loading. Most of these injuries occur during highly dynamic movements with rapid changes in direction. However, no data have been previously presented pertaining to the differences in biceps femoris activation during concentric, eccentric and stretch-shortening movements. The present study is the first study to directly compare muscle activation in a purely concentric movement to a movement that involves the stretch-shortening cycle. The findings of this study suggest muscular injury may be more likely in a purely concentric, braking movement than during a plyometric-type of movement that not only absorbs energy, but releases elastic energy in a coupled concentric contraction.

While this study presents novel findings pertaining to biceps femoris muscle activation during concentric, eccentric and a coupled concentric-eccentric, stretch-shortening cycle, there are some limitations to the current study. A central limitation is the difference in mechanical load associated with the landing task compared to the counter movement jump. The landing task associated with the purely concentric contraction was performed from a height of 0.45 m while the counter movement jump was conducted from standing height. The substantially lower mechanical demand associated with the concentric component of the counter movement jump may underlie the significant differences in muscle activation between the two movements. A second limitation of the present study is that the scope of this study only pertains to muscle activation rather than lower extremity kinetics, muscle forces or tension within the elastic components of the muscle-tendon unit. Further research may investigate muscular compared to elastic contributions to energy dissipation during concentric versus eccentric muscle contractions.

## Conclusion

In conclusion, the stretch-shortening cycle offers many advantages to athletic performance. While previous research has revealed the mechanical and neurological underpinnings of the stretch-shortening cycle, this study revealed that neuromuscular activation is significantly greater in solely concentric or eccentric contractions compared to the stretch-shortening cycle. These differences in muscle activation may be related to muscular injury during deceleration and energy dissipation.
